# Genetic stability of *Aedes aegypti* populations following invasion by *w*Mel *Wolbachia*

**DOI:** 10.1186/s12864-021-08200-1

**Published:** 2021-12-14

**Authors:** Meng-Jia Lau, Thomas L. Schmidt, Qiong Yang, Jessica Chung, Lucien Sankey, Perran A. Ross, Ary A. Hoffmann

**Affiliations:** 1 Pest and Environmental Adaptation Research Group, Bio21 Institute and the School of BioSciences, The University of Melbourne, Parkville, Victoria Australia; 2 Melbourne Bioinformatics, The University of Melbourne, Parkville, Victoria Australia

**Keywords:** *Wolbachia*, *Aedes aegypti*, Evolve-and-resequence, Population replacement, Genome-wide association study

## Abstract

**Background:**

*Wolbachia w*Mel is the most commonly used strain in rear and release strategies for *Aedes aegypti* mosquitoes that aim to inhibit the transmission of arboviruses such as dengue, Zika, Chikungunya and yellow fever. However, the long-term establishment of *w*Mel in natural *Ae. aegypti* populations raises concerns that interactions between *Wolbachia w*Mel and *Ae. aegypti* may lead to changes in the host genome, which could affect useful attributes of *Wolbachia* that allow it to invade and suppress disease transmission.

**Results:**

We applied an evolve-and-resequence approach to study genome-wide genetic changes in *Ae. aegypti* from the Cairns region, Australia, where *Wolbachia w*Mel was first introduced more than 10 years ago. Mosquito samples were collected at three different time points in Gordonvale, Australia, covering the phase before (2010) and after (2013 and 2018) *Wolbachia* releases. An additional three locations where *Wolbachia* replacement happened at different times across the last decade were also sampled in 2018. We found that the genomes of mosquito populations mostly remained stable after *Wolbachia* release, with population differences tending to reflect the geographic location of the populations rather than *Wolbachia* infection status. However, outlier analysis suggests that *Wolbachia* may have had an influence on some genes related to immune response, development, recognition and behavior.

**Conclusions:**

*Ae. aegypti* populations remained geographically distinct after *Wolbachia w*Mel releases in North Australia despite their *Wolbachia* infection status. At some specific genomic loci, we found signs of selection associated with *Wolbachia*, suggesting potential evolutionary impacts can happen in the future and further monitoring is warranted.

**Supplementary Information:**

The online version contains supplementary material available at 10.1186/s12864-021-08200-1.

## Background

*Wolbachia* are bacteria that live inside the cells of many insects and induce important phenotypic effects on their hosts that can be harnessed for pest and disease control. *Wolbachia*-infected *Aedes aegypti* mosquitoes have now been released in multiple locations of the world [1–3] to help reduce the transmission of arboviruses such as dengue, Zika, Chikungunya and yellow fever [4–6]. *Wolbachia w*Mel, which was transferred artificially from *Drosophila melanogaster* into *Ae. aegypti* [6], was first released in Gordonvale and Yorkeys Knob, Queensland, Australia, around a decade ago [7] where it invaded the local population through cytoplasmic incompatibility (CI). CI results in uninfected females less likely to produce viable offspring if they mate with infected males. In contrast, infected females produce viable offspring when they mate with uninfected males or males infected by the same *Wolbachia* strain, and these offspring are infected [8]. This allows *Wolbachia* to invade and be self-sustained in a population but may also increase population divergence because it can reduce the “effective migration rate” [9, 10] between infected and uninfected populations. *Wolbachia* can also impact mitochondrial DNA (mtDNA) variation through indirect linkage disequilibrium [11–13].

With the success of *Wolbachia* in suppressing dengue following invasion [1, 3], many studies have now focused on the sustainability of this approach beyond the initial spread, such as the maintenance of high infection levels [14], fitness costs [15, 16] and evolutionary adaptation [17, 18]. The potential evolutionary changes in *Wolbachia w*Mel-infected *Ae. aegypti* as well as in the bacterial genome itself following releases in the field have raised concerns about the long-term effectiveness of the strategy. The genetic background of the mosquito host can affect the capacity of *Wolbachia* to invade populations and suppress arboviruses [2, 19, 20]. *Aedes aegypti* has a short generation interval of about one month, and so if the introduction of *Wolbachia* triggers an evolutionary process in the mosquito genome this could be observable within a few years after invasion. Evolutionary changes in response to natural *Wolbachia* infections have previously been noted and appear to involve both the *Wolbachia* and host genomes [21, 22], affecting the population dynamics of *Wolbachia* infections. Adaptations can occur to counter any negative fitness effects of *Wolbachia*, as documented in *Drosophila* [21, 23], and negative fitness effects are particularly evident in novel infections transfected into new hosts [24].

There are no published studies investigating evolutionary changes in wild host populations at the genomic level following a *Wolbachia* release. Any putative changes may guide further phenotypic comparisons based on the types of candidate genes identified. However, in *Ae. aegypti*, there are challenges in characterizing genome changes after *Wolbachia w*Mel infection. First, the genome of *Ae. aegypti* contains a large proportion (47%) of transposable elements (TEs), which result in a large genome size (1.38Gb) compared to other mosquitoes [25–27]. TEs might also enhance rates of evolution, given that they are involved with gene regulation, and increase genome plasticity [28]. Moreover, other environmental factors in field-collected samples may be important, and the impacts of gene flow following the activity of *Wolbachia* release will increase the difficulty of outlier analysis. Finally, compared to model organisms, the genome of *Ae. aegypti* is still relatively poorly annotated, with only 256 proteins (< 1%) reviewed in the Swiss-Prot database (https://www.uniprot.org/taxonomy/7159).

In this study, we analyze pooled whole genome sequencing (WGS) data of mosquitoes from Gordonvale, Australia, where releases first took place, covering three different time points from the pre- and post-release phase. As a comparison, we also sequenced samples from the nearby Cairns area in Edge Hill, Redlynch and Yorkeys Knob, where *Wolbachia* replacement happened at different times across the last decade. In these locations, the population released involved a *w*Mel transinfected strain that had been repeatedly backcrossed to a Cairns field population background, with the expectation that the released background would be 99.9% Cairns [7]. For releases in Yorkeys Knob and sites around Cairns, we did not expect the release population to differ from the background population because there is movement of mosquitoes around this area as reflected by the occasional detection of *Wolbachia*-infected mosquitoes after the release [7]. On the other hand, Gordonvale is a relatively isolated population which may have its own seasonal dynamics. We combined analyses of temporal and spatial variation in *Wolbachia* infection status to reveal genetic diversity in the mosquito populations and the potential impact of *Wolbachia w*Mel on the genome of its host *Ae. aegypti*.

## Results

### Genetic variation in *Aedes aegypti* populations

**Fig. 1:**
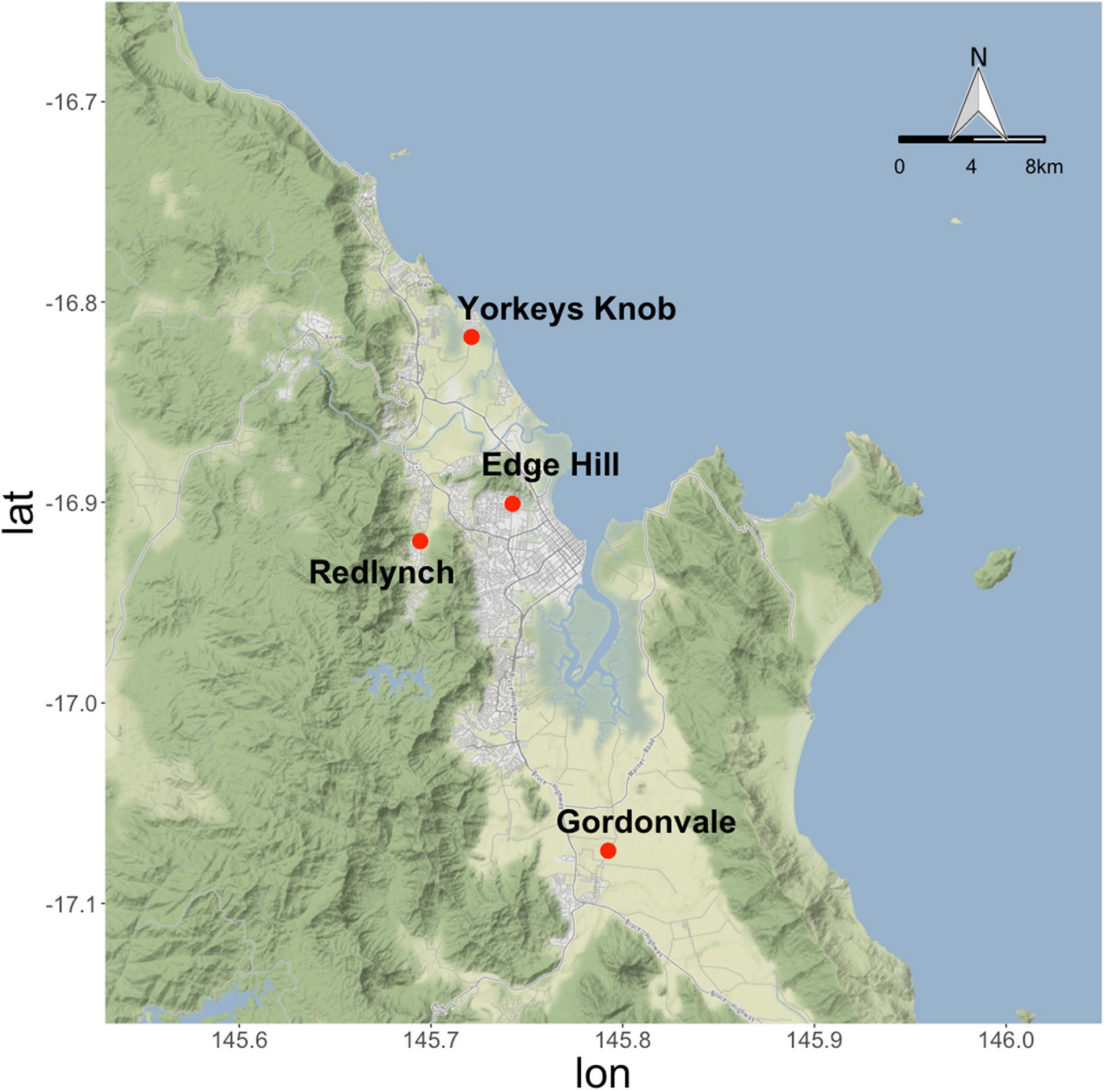
Locations of sampled *Aedes aegypti* populations. Samples from Gordonvale were collected in 2010, 2013 and 2018, while samples from other locations were collected in 2018. Axes show longitude (lon) and latitude (lat). The map is derived from ggmap package in R, interface to Google Maps application program interface (API) (https://developers.google.com/maps/)

**Fig. 2:**
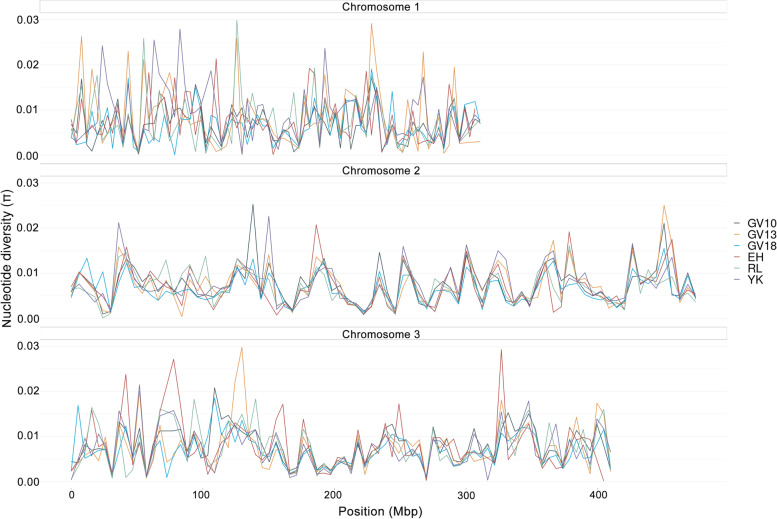
LOESS-smoothed curves of genome-wide nucleotide diversity (π). Six populations of *Ae. aegypti* measured in 10 kbp non-overlapping windows. GV10 and GV13 represent samples collected in 2010 and 2013 from Gordonvale; GV18, EH, YK and RL represent samples collected in 2018 from Gordonvale, Yorkeys Knob, Edge Hill and Redlynch respectively

*Aedes aegypti* were collected from four sites around Cairns, Australia, at different times pre- and post-*Wolbachia* releases (Fig. 1, Table 1). We investigated patterns of genetic variation within populations and obtained Tajima’s pi (nucleotide diversity π) for each population at 10 kbp non-overlapping windows. Differences in π among populations depended on chromosomal location. Near the center of each chromosome, diversities were low and similar for all populations (Fig. 2). In areas far from the centromere, variation between populations was large. This pattern has been observed in previous sequencing studies [27, 29], which may result from a high proportion of TEs and satellites in non-coding regions. Overall, nucleotide diversity was highest on chromosome 1, which contains the sex determining locus and contains relatively lower gene densities and TEs but higher satellites compared to chromosomes 2 and 3 [30].

**Table 1:** Summary of *Aedes aegypti* collections and designations of samples used in comparisons

Population name	Collecting year	Location	*Wolbachia* infection (Y/N)	Year of population replacement	Sample size
GV10	2010	Gordonvale	N	2011	56
GV13	2013	Gordonvale	Y	2011	51
GV18	2018	Gordonvale	Y	2011	52
EH	2018	Edge Hill	Y	2013	52
RL	2018	Redlynch	N	2019	52
YK	2018	Yorkeys Knob	Y	2011	52

**Fig. 3:**
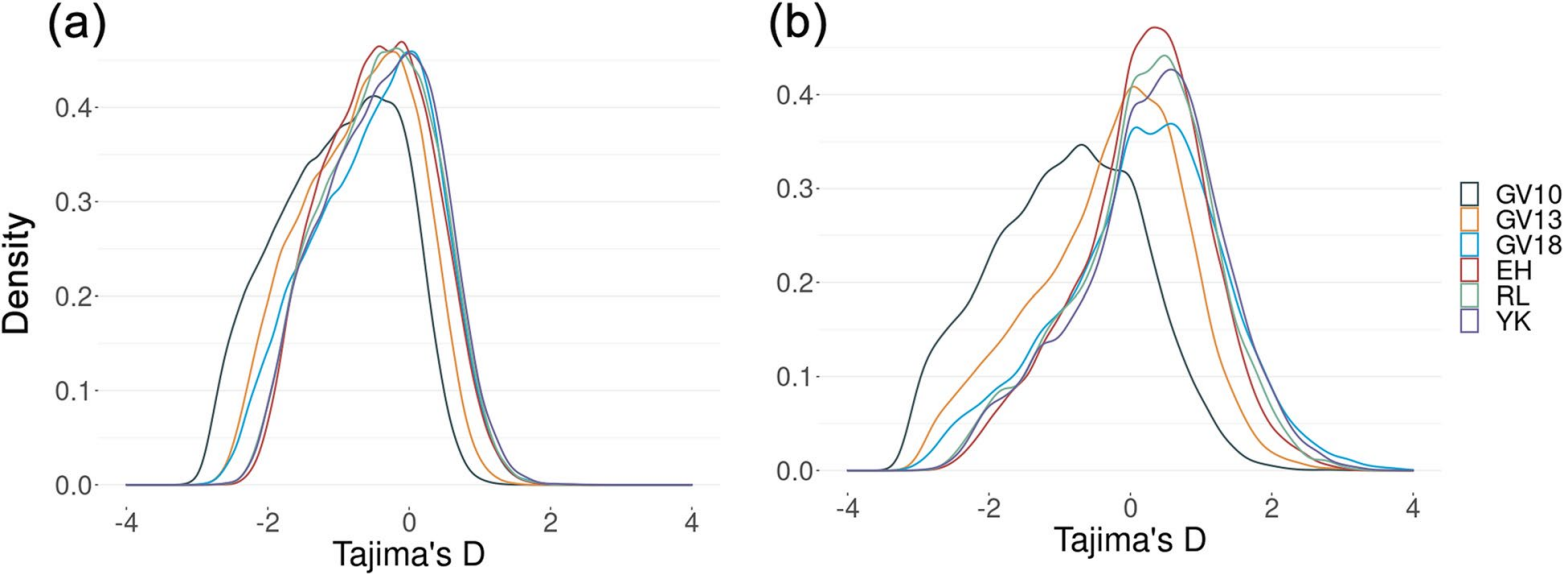
Density distributions of Tajima’s D values. Tajima’s D measured at (**a**) the genome level measured in 10 kbp non-overlapping windows and at (**b**) the gene level. GV10 and GV13 represent samples collected in 2010 and 2013 from Gordonvale; GV18, EH, YK and RL represent samples collected in 2018 from Gordonvale, Yorkeys Knob, Edge Hill and Redlynch respectively

We calculated Tajima’s D for each population at 10 kbp non-overlapping windows and at the gene level. We found that the density distributions of values were similar between populations, except for the Gordonvale pre-release (GV10) sample which shows a high proportion of negative values (Fig. 3). This pattern is more obvious at the gene level (Fig. 3b) than at the genome level measured in 10 kbp non-overlapping windows (Fig. 3a), Additional file 1). The four 2018 populations converge regardless of their *Wolbachia* infection status or time since *Wolbachia* was invaded. This suggests that the pattern reflects a difference in GV10 before release rather than an effect of *Wolbachia* per se.

### Geographic segregation of *Aedes aegypti* populations

We investigated genetic distances from the average of pairwise Fst (Fixation index) values through 100 kbp non-overlapping windows. The temporally-separated Gordonvale samples tended to have lower pairwise Fst than population pairs from different locations (Table 2). For isolation by distance (IBD) test, we also obtained a geographic distance matrix with the four 2018 populations, which was built based on the natural log transformation of the shortest road distance between the sampled locations (Table 2). Genetic distance had a weak but non-significant correlation with natural log transformed geographic distance in a Mantel test (r = 0.66, *p* = 0.12).

**Table 2:** Matrix of genetic distance (above diagonal) and geographic distance (below diagonal)

	GV10	GV13	GV18	EH	RL	YK
GV10	0	0.045	0.040	0.056	0.059	0.064
GV13	0	0	0.057	0.063	0.067	0.068
GV18	0	0	0	0.052	0.057	0.059
EH	3.243	3.243	3.243	0	0.051	0.054
RL	3.547	3.547	3.547	2.526	0	0.057
YK	3.694	3.694	3.694	2.821	2.773	0

**Fig. 4:**
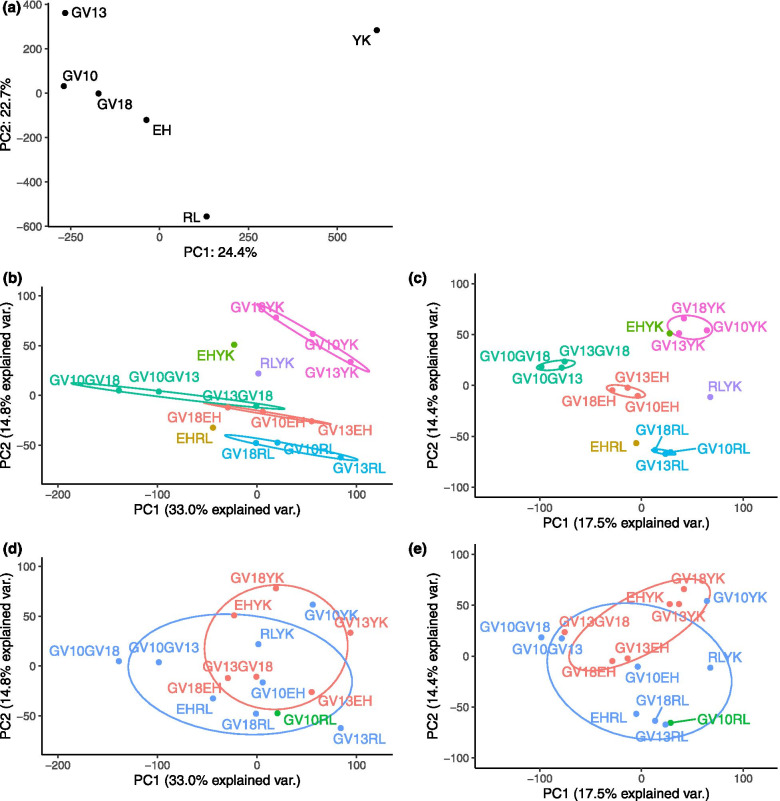
Principal components analysis based on MAF or pairwise Fst. PCA plots of (**a**) allele frequency of *Aedes aegypti* samples (MAF > 0.1, minimal coverage > 50); (**b**, **d**) pairwise Fst throughout the genome with 100 kbp non-overlapping windows, and (**c**, **e**) pairwise Fst within genes. Colors in (**b**, **c**) represent geographical comparisons. The blue color in (**d**, **e**) represents comparisons between *Wolbachia*-infected and uninfected samples, while red represents comparisons within *Wolbachia*-infected samples and green represents the comparison within uninfected samples. The 95% confidence ellipses show data clustering

There were 461,067 single-nucleotide polymorphisms (SNPs) left after filtering with minimal depth of 50 in all populations and an average minor allele frequency (MAF) > 0.1. A principal components analysis (PCA) based on these SNPs showed the first two PCs accounted for 24.2 and 22.9% of the variance respectively (Fig. 4a). The three temporally-separated Gordonvale samples fell out together, but GV10 was closer to GV18 than to GV13. We also ran PCAs on pairwise Fst differences in 100 kbp non-overlapping windows across the genome (Fig. 4b, d) and at the gene level (Fig. 4c, e). When testing the similarity of infected or uninfected populations (Fig. 4d, e), we found little evidence for any clustering of populations related to *Wolbachia* infection status either across the genome or at the gene level. On the other hand, these populations clustered geographically (Fig. 4b,c). Comparisons between the Gordonvale samples and Yorkeys Knob, Edge Hill, and Redlynch showed consistent clustering by location, and stronger clustering at the gene level (Fig. 4c) than across the genome (Fig. 4b).

### Bayesian models to identify outliers potentially associated with *Wolbachia*

To investigate potential selection associated with *Wolbachia*, we used 461,067 SNPs from the above filtering process and used two *Bayesian* models from BayPass v. 2.2 [31] for *Wolbachia*-related outlier analysis.

**Fig. 5:**
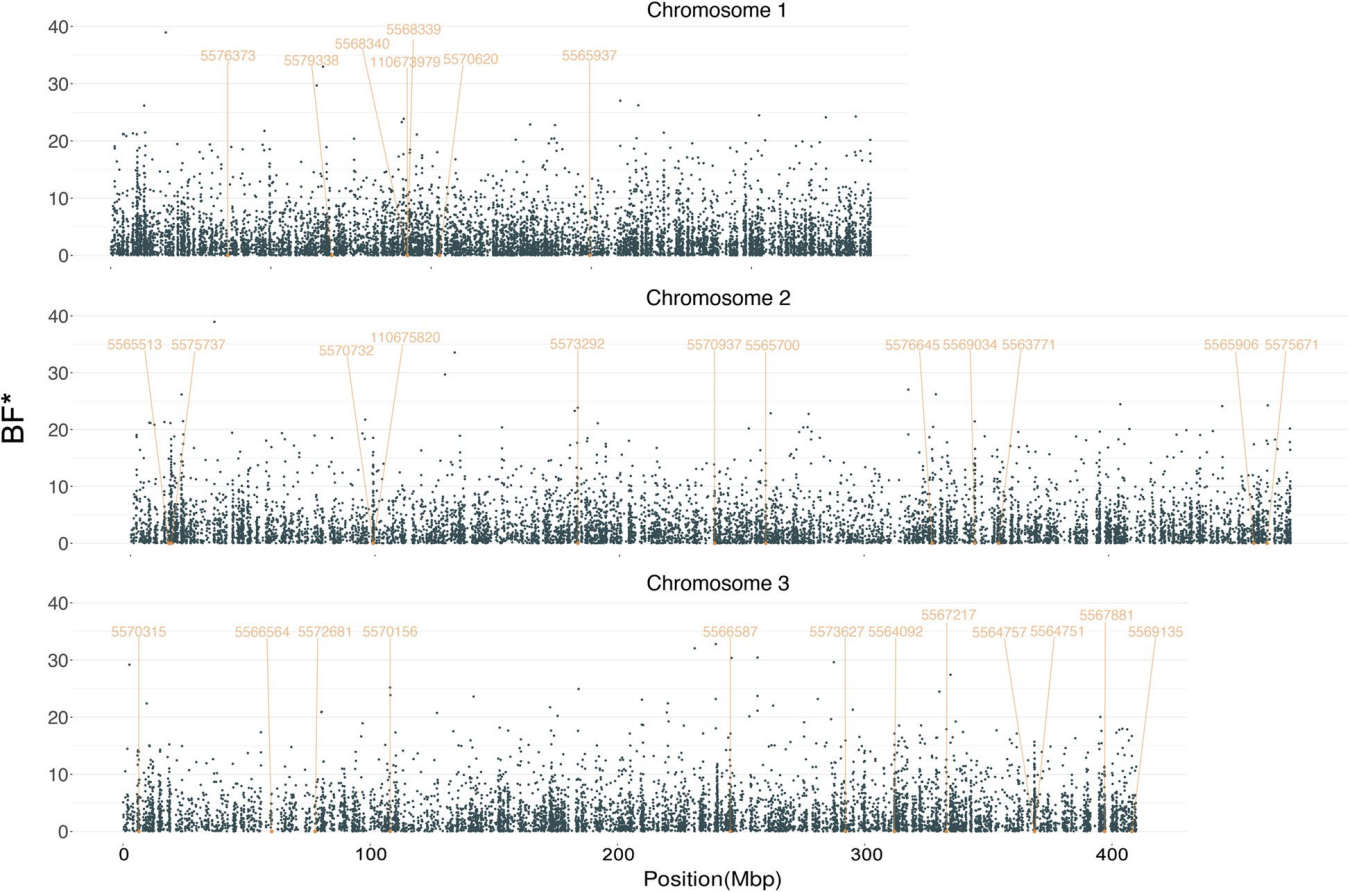
Manhattan plot of SNPs in covariate model representing *Wolbachia* infection status. Points represent SNPs with a positive Bayes Factor (BF) in covariate model. Orange labels represent genes and their positions associated with “strongly” outliers that identified from the combination of two Bayesian models

In the covariate model of BayPass, with *Wolbachia* infection status in the comparison, we found 2415 SNPs showing a “substantial” signature of selection with an average BF* (Bayes Factor (BF) in dB units) > 5, and 391 showing “strongly-selected” signature of selection with an average BF* > 10 (Fig. 5).

The introduction of linear relationships in the covariate model, however, can result in a high false positive rate from sampling noise, particularly when large environmental effects are involved and small number of populations are compared. We therefore used a second model to calculate X^T^X, which was analogous to Fst [31, 32], then combined these two models to identify outliers potentially associated with *Wolbachia* infection.

We found that 950 (prior probability: 0.74 in each Mbp) of the “substantial” SNPs fell into the intersection of top 10% X^T^X values in the comparisons between GV10 and GV13 and between GV10 and GV18, including 229 SNPs distributed on chromosome 1, 390 on chromosome 2 and 330 on chromosome 3, while one was found on an assembly scaffold NW_018735222.1. A proportion of these SNPs were concentrated in specific regions, with posterior probability at least five times greater than prior probability (> 3.7 in each Mbp), suggesting selection due to *Wolbachia* (Table 3). These SNPs were considered as “substantial” outliers associated with the *Wolbachia* infection.

**Table 3:** Genomic regions potentially under *Wolbachia w*Mel selection

Chromosome	Position	Size (Mbp)	Number of SNPs
1	96,900,000-105,500,000	8.6	39
1	138,000,000-140,500,000	2.5	13
1	148,500,000-149,500,000	1.0	11
1	183,000,000-188,300,000	5.3	40
1	203,100,000-206,200,000	3.1	22
2	15,600,000-16,800,000	1.2	27
2	20,700,000-21,200,000	0.5	11
2	98,800,000-99,300,000	0.5	18
2	345,200,000-345,400,000	0.2	19
2	396,100,000-396,500,000	0.4	15
3	239,700,000-249,400,000	9.7	31
3	311,900,000-316,000,000	4.1	25
3	368,400,000-368,600,000	0.2	14

We also used a stricter criterion: average BF* > 10 and falling into the intersection of top 5% X^T^X values in the GV10, GV13 comparison and the GV10, GV18 comparison. We then found 113 SNPs that were highly associated with *Wolbachia* infection and were considered as “strong” outliers.

### Pathway analysis and gene ontology enrichment analysis

The “substantial” outliers were distributed within 187 genes (Additional file 2). When a region 50 kbp upstream and 50 kbp downstream around each outlier was considered, this number increased to 1436 genes of interest (Additional file 3). We performed a pathway analysis of these 1436 genes through the KEGG database [33] and found eight pathways significantly involved (Table 4, Additional file 4), including pathways involved with development and immune response (MAPK signaling pathway and Toll and Imd signaling pathway). We also identified homologues of these genes in *D. melanogaster* for the gene ontology (GO) enrichment analysis, 108 gene sets were significantly enriched in biological process (Additional file 5), 42 in cellular component (Additional file 6) and 22 in molecular function (Additional file 7). In general, we found that *Wolbachia w*Mel may modulate genes with diverse functions such as cell development, interaction, cellular response, cellular transport, neurogenesis, lipid and glucide metabolization, behavior and immune response.

**Table 4:** Significantly enriched pathways in KEGG database potentially involved with *Wolbachia w*Mel infection (copyright permission 210,807) [33]

Pathway	Description	Gene Ratio	Bg Ratio	*P* value
aag00232	Caffeine metabolism	6/296	12/3281	< 0.001
aag00981	Insect hormone biosynthesis	9/296	40/3281	0.008
aag00052	Galactose metabolism	7/296	30/3281	0.015
aag04341	Hedgehog signaling pathway - fly	6/296	25/3281	0.021
aag04013	MAPK signaling pathway - fly	14/296	92/3281	0.034
aag00230	Purine metabolism	14/296	96/3281	0.047
aag00270	Cysteine and methionine metabolism	6/296	30/3281	0.048
aag04624	Toll and Imd signaling pathway	9/296	54/3281	0.050

The “strong” outliers were distributed across 31 genes (Fig. 5, Additional file 1, Additional file 8). These included gene 5,564,751, which encodes cytochrome P450, which can be enriched in response to dengue virus infection in refractory mosquitoes [34] and is associated with insecticide resistance [35, 36]. Carbonic anhydrase, encoded by gene 5,565,700, has the function of balancing pH in mosquito midgut [37, 38]. Gene 5,569,135 encoding ecdysone protein E75, and gene 5,572,681 encoding lipophorin, have been highly expressed in females after blood feeding and are potentially involved in regulation of oogenesis and vitellogenesis [39, 40]. Charged multivesicular body protein, encoded by gene 5,573,292, is associated with endosome formation and can influence mosquito immune response [41].

## Discussion

We show that *Ae. aegypti* populations in Cairns remain geographically distinct following releases of *Wolbachia w*Mel, but also find some evidence suggesting evolutionary change in mosquito populations. When interpreting these results, it is important to consider the release process and which populations were targeted, and the fact that *Wolbachia*-induced CI can increase population divergence by reducing the migration rate across host populations when only one or both (in the case of different *Wolbachia* strains) are infected [9, 10]. In release areas around Cairns, Gordonvale is relatively isolated from other release locations. Although these populations are not genetically isolated based on microsatellite and EPIC markers [42], they do appear to be somewhat separated based on the SNP markers used in the current study. This may account for the pattern noted for Tajima’s D where the GV10 population was a clear outlier.

Following invasion by *Wolbachia*, there is not only complete replacement of the uninfected mosquito population by *Wolbachia*-infected mosquitoes but also replacement of the mtDNA that hitchhikes along with the *Wolbachia* [12]. Also, while any linkage disequilibrium between the *Wolbachia* and nuclear DNA variants is expected to break down relatively quickly [43], new alleles may nevertheless be introduced into the population. The nuclear DNA constitution of the population might be expected to become more like the release stock for a period as released females and their offspring mate with released and resident males, although local selection should then lead to populations becoming more like the original population. In our case, the genetic similarity among the Gordonvale samples before and after releases might reflect local selection and ongoing introgression of the release stock with the resident population, as GV10 and GV18 are closer than GV13 in the PCA analysis. Furthermore, Yorkeys Knob and Edge Hill remain distinct from each other despite previously being invaded by the same release stock [3, 7].

Populations were more differentiated at the gene level than at the genome level, which may be a consequence of a large proportion (47%) of TEs and satellites associated with non-coding regions [25, 26]. These TEs and satellites were masked in our reference genome [27], resulting in low coverage in sliding windows and were therefore filtered out in the gene analyses. This may explain why there was a high level of variation in the genome-wide scans of π and Tajima’s D. For chromosome 1, which contains the sex-determining regions, pools of male and female individuals were aligned to the reference genome, which can also result in low coverage of windows and induce high levels of genomic variation. Genic regions will be easier to track into the future for further analysis.

When considering the impacts of *Wolbachia* on the *Ae. aegypti* genome, selection in response to local conditions and the impact of *w*Mel on *Ae. aegypti* may only influence patterns of genetic differentiation at specific loci [19]. Our Bayesian outlier analysis identified several regions in each chromosome and genes related to immune response, development, recognition and behavior that may have been under selection. When overlapping these results with previous Tajima’s D analysis, we identified only a small proportion of genes potentially related to selective sweeps, which were mainly distributed on chromosome 2. These potential evolutionary impacts of *Wolbachia w*Mel on the genome of *Ae. aegypti* in the field suggest that further monitoring is warranted, although at this stage other factors unrelated to *Wolbachia* appear to have a larger impact on genomic differentiation among samples.

We found signs of selection on Toll and Imd signaling pathways in KEGG analysis; these are important pathways in immune system [44, 45] and virus blocking processes [45–47]. Previous transcriptomic studies showed up-regulation of these pathways in both *w*Mel and *w*MelPop-infected *Ae. aegypti* [48, 49]. Genes associated with virus blocking are mainly distributed on chromosome 1, in addition to genes associated with cytoskeleton, cell-cell adhesion and signal transduction [19]; these genes also showed up in our GO enrichment analysis. Other than virus blocking, caffeine metabolism was strongly impacted, which may impact hormone metabolism and detoxification when cytochrome P450 is involved [50, 51]. We also detected enriched pathways involved with development, such as insect hormone biosynthesis and the Hedgehog signaling pathways. In the GO enrichment analysis these were represented in cell growth, structure, recognition and behavior.

In the past decade, the *w*Mel infection itself has not evolved in terms of either sequence composition or density since establishment in *Ae. aegypti* in northern Queensland, Australia [52]. Phenotypic comparisons also suggest limited changes in host fitness costs and CI since population replacement in this region [14, 18], although the number of fitness-related traits scored so far has been limited. Blockage of virus transmission also appears stable to date [53, 54], and may persist through ongoing selection favoring high viral blocking in *Ae. aegypti* populations [19]. However, the outlier loci detected here suggest that ongoing monitoring of phenotypic effects is warranted.

## Conclusions

*Wolbachia w*Mel-infected *Ae. aegypti* mosquitoes have been released successfully in the field to help reduce the transmission of arboviruses, but interactions between *w*Mel and *Ae. aegypti* could result in adaptation [55, 56], altering virus blocking efficiency [19, 57], host fecundity [21] and insecticide resistance [58]. In this study, we have identified *Ae. aegypti* populations as being geographically distinct despite their *Wolbachia* infection status. However, selection associated with *Wolbachia w*Mel may still have influenced variation at some loci. This is the first time that genome evolution associated with *Wolbachia* infection has been examined in field populations where there has been a deliberate release. However, it is hard to draw conclusions about long-term impacts of *Wolbachia* on the mosquito genome, which may take more time to develop, and which may be different in regions where dengue is endemic, unlike in Australia. Our findings highlight the possibility that the effect of *Wolbachia* can interact with the host genomic background, which has been shown previously in phenotypic assays of the longevity effects of *w*MelPop in *Drosophila* [59].

## Methods

### Samples and study sites

*Aedes aegypti* samples were collected from four sites around Cairns (Fig. 1, Table 1) [60]. In Gordonvale, samples were collected three times: in the summer of 2010 (pre-release), as well as in 2013 and 2018 (2 and 7 years post-release given that the area was stably invaded in 2011 [7]). Samples from Yorkeys Knob, Edge Hill and Redlynch were collected in 2018. Yorkeys Knob experienced *Wolbachia* invasion at the same time as Gordonvale in 2011, and Edge Hill which was invaded in 2013 [61]; Redlynch was an uninfected area when sampled in 2018. Gordonvale samples from 2010 and 2013 were collected by BG-Sentinel traps (Biogents, Regensburg, Germany) while 2018 samples were collected by ovitraps, taking care to sample only 1-2 larvae per ovitrap to reduce the likelihood of siblings being sampled [62]. Samples from Gordonvale 2010 (GV10) and 2013 (GV13) were stored in absolute ethanol at the adult stage while samples collected from 2018 (GV18, EH, YK and RL) were stored in absolute ethanol at the fourth instar larval stage.

### DNA extraction and library preparation

Whole genomic DNA was extracted from each individual mosquito using Qiagen DNA Blood and Tissue kit (Venlo, Limburg, NL) for 2010 and 2013 samples, and using Roche High Pure™ PCR Template Preparation Kits (Roche Molecular Systems, Inc., Pleasanton, CA, USA) for 2018 samples. *Wolbachia* infection status was confirmed by a diagnostic qPCR test as outlined elsewhere [63]. The concentration of extracted individual DNA was measured using Quantitation Qubit™ 1X dsDNA HS Assay Kit (Invitrogen Life Technologies, USA). Samples from each of the six populations (Table 1) were pooled prior to sequencing based on an equal amount of DNA from each individual. Each population was sent for whole genome sequencing with > 50 depth via Illumina Hiseq2500 using 100 bp paired read chemistry for GV10 and GV13, and 150 bp paired read chemistry for GV18, EH, YK and RL libraries.

Raw sequences were trimmed using Trimmomatic v. 0.39 to truncate and remove low quality reads by requiring all reads to have all bases with a phred score > 20 and read length > 70 bp. The reference genome AaegL5.0 [27] was indexed and reads were aligned using bowtie2 v. 2.3.4.3 with the --very-sensitive-local preset [64], with alignment rates ranging from 79.3 to 84.0%. Samtools v.1.9 was used with default parameters to sort, mark and remove duplicates and generate pileup files requiring a minimal mapping quality of 20.

### Estimation of genetic variation

We investigated patterns of genetic variation within populations using PoPoolation v. 1.2.2 [65] with the genomic annotation file from the reference AaegL5.0 which has TEs and satellites masked. We calculated Tajima’s pi (nucleotide diversity π) for each population at 10 kbp non-overlapping windows and Tajima’s D for each population at 10 kbp non-overlapping windows and at the gene level with a minimum coverage of 20. Windows with low coverage generated no values and were omitted before adjusting the shape of lines across each chromosome by a LOESS smooth curve [66]. Within each retained window, at least 60% of sites had sufficient coverage (Additional file 9), indicating the high quality of all windows used for downstream analysis. The value of Tajima’s D was calculated from allele frequencies in selected regions and was used to detect directionality of selection. Under a standard neutral model with no change in population size, a strongly negative Tajima’s D value can indicate directional selection removing variation, while a strongly positive value can indicate balancing selection maintaining variation, with 0 reflecting an absence of selection.

For genetic variation between populations, we used PoPoolation2 v. 1.201 [67] to obtain allele frequency differences for each SNP. The SNPs were filtered to have a coverage > 50 in all populations, ensuring that poorly sequenced areas were excluded. We also filtered to ensure an average MAF > 0.1 [68]. We also obtained pairwise Fst values for non-overlapping 100 kbp windows and for gene sets. The PCA was generated by the prcomp function and the ggbiplot package in R [69] based on: 1) MAF across the SNPs after initial filtering as mentioned above; 2) pairwise Fst values from 100 kbp non-overlapping windows to indicate genetic distance patterns across the genome (genome level) [70]; and 3) pairwise Fst for genes (gene level). These Fst estimates were then used to assess patterns of similarity among samples with the same *Wolbachia* infection status in a pairwise comparison, and the same geographic distance in a pairwise comparison.

We further investigated IBD patterns among 2018 samples from the four locations by computing Fst* = Fst/(1 − Fst) [71] based on the average pairwise Fst values from 100 k bp non-overlapping windows. A geographic distance matrix was built based on the natural log transformation of the shortest road distance between the sampled locations as mosquito movement would be mostly by road transport [72]. We then looked for Fst patterns that might be related to this distance measure and ran a Mantel test through the ade4 package in R to test the relationship between genetic distance and geographic distance [73], with only 2018 populations included.

### Identification of outliers potentially associated with *Wolbachia*

We used two models from a *Bayesian* outlier *approach* BayPass v. 2.2 [31] to identify outliers associated with *Wolbachia* infection. Firstly, we used a standard covariate model [31, 74], which requires a file providing values of each covariate to produce the Bayes factor (BF), the ratio of the likelihood of posterior and prior hypotheses, which can quantify the relative evidence of a candidate SNP being under selection [75]. BF were exported in dB units (BF^*^ = 10 × log10(BF)); the association with environmental variance was considered as “substantial” when BF* > 5, “strongly-selected” when BF* > 10 and “decisively-selected” when BF^*^ > 13 following H Jeffreys [75]. We modelled *Wolbachia* infection status as a binary covariate by setting each infected sample as 1 and each uninfected sample as 0. The measures of BF values are based on an Importance Sampling Approximation, which is unstable for single runs in particular when the number of population is small, so we averaged the BF values of three runs with different seeds from the random number generators following the suggestion in the manual of BayPass [31]. The introduction of linear relationships in this model, however, can lead to a high false positive rate from sampling noise, as the relationships of allele frequency among multiple populations are also influenced by other environmental factors, and are not independent from each other [32]. Based on this model, we generated plots for SNPs with average BF* > 0 to illustrate the potential effects of the *Wolbachia* infection at the genome level; SNPs that could not be assigned a position on one of the three autosomes were discarded.

Secondly, we used a BayPass core model to identify outliers from comparisons between GV10 and GV13, GV10 and GV18, given that Gordonvale was the only location where we had samples at time points before and after the release. The infection of *Wolbachia* was considered as the main variable across time. An X^T^X algorithm approach was used in this model, which was analogous to an Fst comparison [31, 32]. The X^T^X value was used to identify selection pressure, with higher values suggesting positive selection and smaller values suggesting balancing selection [32]. In this model, we considered SNPs with X^T^X values greater than the 90% threshold or 95% threshold in both the GV10 and GV13 comparison and the GV10 and GV18 comparison as candidates for intersecting with the “substantial” SNPs and “strongly-selected” SNPs from the first analysis. However, this model is unable to exclude the noise from gene flow or genetic drift due to the lack of duplicates, which can also cause false positives. We therefore considered the intersection of outliers from the above two models as SNPs potentially associated with *Wolbachia* infection. These SNPs were then matched with the GTF annotation file from NCBI to obtain a list of outlier genes.

### Pathway analysis and gene ontology enrichment analysis

We considered SNPs from the intersection of the two BayPass models above as outliers potentially under selection and searched the open reading frames to obtain a list of potentially important genes within a 100 kbp region around each SNP (50 kbp upstream and 50 kbp downstream) [76]. These genes were then searched through the KEGG pathway database [33] for interpretation. We then used BLAST+ (version 2.9.0) with the UniProtKB/Swiss-Prot database [77] to identify homologous proteins in *D. melanogaster*. Only best matches were retained and further filtered with an e-value cut off 1.0E-10 and > 60% identity [78, 79]. Gene ontology (GO) enrichment analysis was then undertaken using the R packages clusterProfiler [80] and DOSE [81], with a false discovery rate cut-off of 0.05 after multiple hypothesis testing correction.

## Supplementary Information

**Additional file 1.**

**Additional file 2.**

**Additional file 3.**

**Additional file 4.**

**Additional file 5.**

**Additional file 6.**

**Additional file 7.**

**Additional file 8.**

**Additional file 9.**

## Data Availability

Aligned and merged sequencing data for *Aedes aegypti* used in this study are available from NCBI Sequence Read Archive (SRA), under the BioProject ID PRJNA776956. All data generated during this study are included within this article and its additional files.
