# Supplementary material for: Genetic stability of Aedes aegypti populations following invasion by wMel Wolbachia
Source: BMC Genomics. 2021 Dec 14;22:894. doi: 10.1186/s12864-021-08200-1 (PMC8670162; doi:10.1186/s12864-021-08200-1)
Supplement: Supplementary file 1 — Additional file 1. [file 12864_2021_8200_MOESM1_ESM.docx]

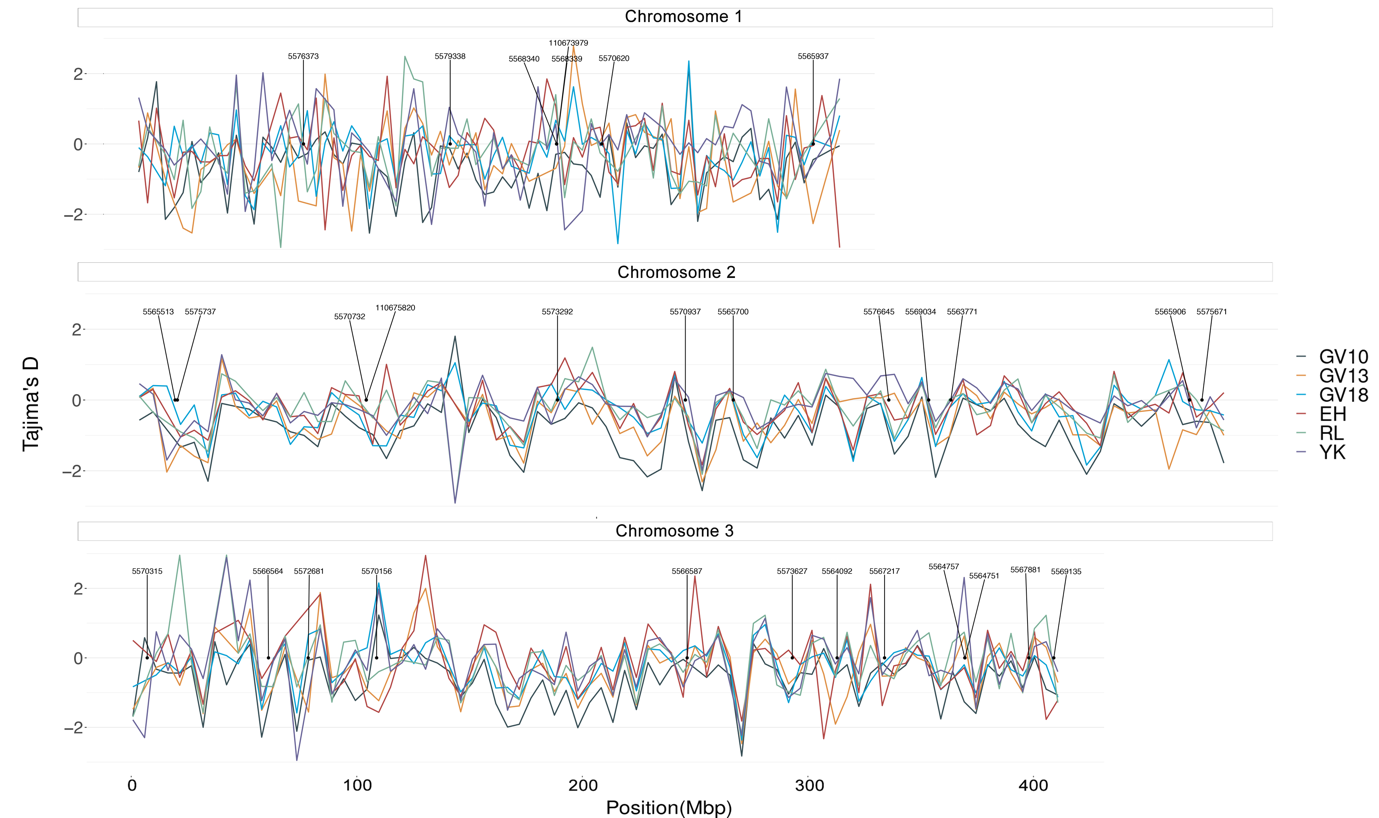


Additional file 1. LOESS-smoothed curves of Tajima’s D. Six populations of Ae. aegypti measured in 10 kbp non-overlapping windows. GV10 and GV13 represent samples collected in 2010 and 2013 from Gordonvale; GV18, EH, YK and RL represent samples collected in 2018 from Gordonvale, Yorkeys Knob, Edge Hill and Redlynch respectively. Orange labels represent genes and their positions associated with “strongly” outliers that identified from the combination of two Bayesian models in the outlier analysis.
