# Supplementary material for: Genetic stability of Aedes aegypti populations following invasion by wMel Wolbachia
Source: BMC Genomics. 2021 Dec 14;22:894. doi: 10.1186/s12864-021-08200-1 (PMC8670162; doi:10.1186/s12864-021-08200-1)
Supplement: Supplementary file 9 — Additional file 9. [file 12864_2021_8200_MOESM9_ESM.docx]

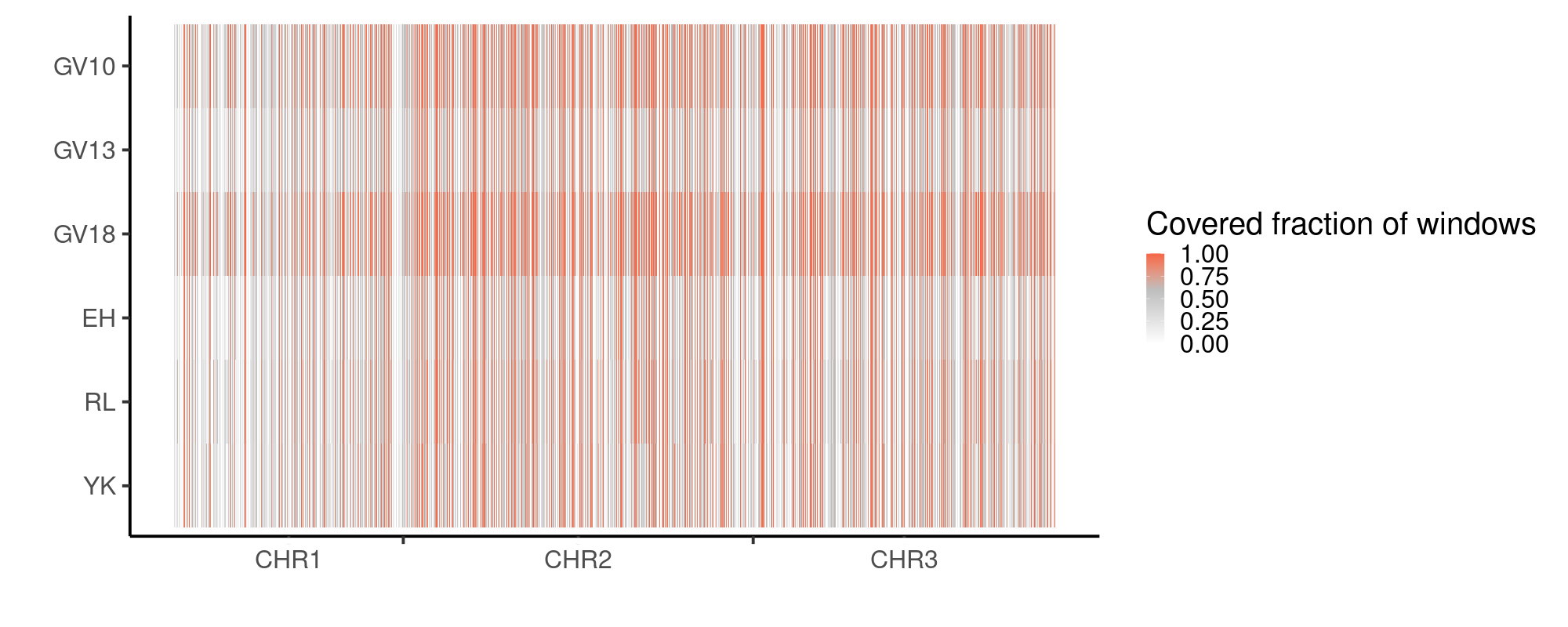


Additional file 9. Covered fraction of each 10 kbp non-overlapping window. Only windows marked with orange color were retained in further Tajima’s pi and Tajima’s D analysis.
